# Seizure-Induced Hemoptysis in a Pediatric Patient

**DOI:** 10.1155/2022/6059007

**Published:** 2022-12-23

**Authors:** Ritika Nangia, Margaret Kahwaty, Ashutosh Sachdeva, Nidhi Kotwal

**Affiliations:** ^1^Section of Interventional Pulmonology, Division of Pulmonary and Critical Care, University of Maryland School of Medicine, Baltimore, MD 21201, USA; ^2^Department of Pediatrics, University of Maryland, Baltimore 22S Greene Street, Baltimore, MD 21201, USA; ^3^Division of Pulmonology and Allergy, Department of Pediatrics, University of Maryland, 737 W Lombard Street, Ste 314, Baltimore, MD 21201, USA

## Abstract

Hemoptysis can complicate seizures, albeit rarely. This unfamiliar presentation, reported infrequently in adults, can also affect children. This remains a rare clinical entity in pediatrics and we report one such case and its association with sterol carrier protein (SCP) gene mutation. We present a case of a 16-year-old male with recurrent episodes of hemoptysis following seizures. The diagnostic workup for etiology of the hemoptysis was unrevealing and he was ultimately treated for neurogenic pulmonary edema as a diagnosis of exclusion. He achieved complete resolution with supportive care and diuretics. Our case report describes the clinical and radiological presentation and overall management of post-ictal pulmonary hemorrhage and edema in a pediatric patient. In addition, it reports a new finding of possible association with sterol carrier protein (SCP2) carrier status. It also highlights a rare but potentially life-threatening consequence of inadequate seizure control in pediatric patients.

## 1. Introduction

Hemoptysis is infrequent in children and is usually secondary to infection, foreign body aspiration, tracheostomy complications, or congenital heart disease [1]. Hemoptysis as a complication of seizures is rare and remains a diagnosis of exclusion. Diffuse alveolar hemorrhage (DAH) following generalized tonic-clonic seizures has been reported in literature, with age range of cases being 16–41 years [2]. The authors of reference [3] reported that cases in pediatric literature remain sparse [4] and there is a need for improved understanding of clinicopathological associations of this rare entity. We report a pediatric case of alveolar hemorrhage following generalized tonic-clonic seizure and an association with a genetic mutation previously unreported in English literature [2–4].

Our patient was ultimately diagnosed with neurogenic pulmonary edema as the etiology for his recurrent hemoptysis in the setting of seizures. First reported by Shanahan in 1908 [5], neurogenic pulmonary edema is believed to be a complex interplay of elevated intravascular hydrostatic pressure and pulmonary capillary leak in conjunction with possible structural damage to the alveolar-capillary interface [6]. High central sympathetic drive secondary to raised intracranial pressure extending into the post-ictal period contributes to systemic and pulmonary vasoconstriction, likely leading to the pulmonary edema seen in our patient. During admission, he was also found to be a carrier for SCP2, a peroxisomal enzyme with thiolase activity that is required for the breakdown of branched-chain fatty acids. Mutation in the SCP2 gene can cause leukoencephalopathy with dystonia and motor neuropathy due to accumulation of the branched-chain fatty acid pristanic acid in plasma [7]. Its association with seizures, though, has been less clearly defined in literature.

## 2. Case Presentation

**Figure 1:**
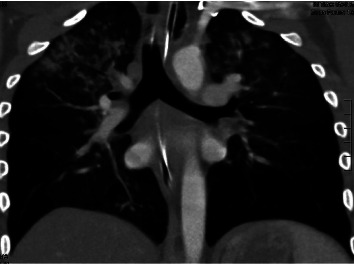
Coronal view of CT chest scan showing diffuse alveolar ground-glass and nodular opacities prior to initiation of diuretic therapy.

**Figure 2:**
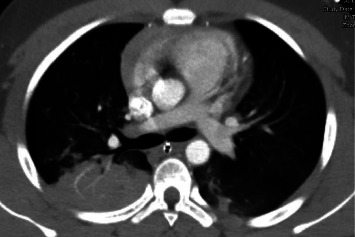
Axial view of CT chest scan showing bilateral alveolar ground-glass opacities with right upper lobe posterior segment consolidation.

A 16-year-old male presented to the emergency department alone with initial complaint of “homelessness.” Shortly after, he collapsed and had a witnessed, generalized tonic-clonic seizure that lasted about five minutes. He did not meet the criteria for status epilepticus. On examination, he had blood in his mouth without any visible trauma and an oxygen saturation of 72% on room air. He was promptly intubated for airway protection, and approximately, 10 mL blood was subsequently suctioned from the endotracheal tube. He was admitted to the pediatric intensive care unit (PICU) for further management. Extensive investigative workup was initiated. Bloodwork demonstrated white blood cell (WBC) count, 8300 K/mcL, hemoglobin, 13.9 g/dL, platelets (Plt), 222 K/mcL, C-reactive protein (CRP) < 0.5 mg/dL, erythrocyte sedimentation rate (ESR), 9 mm/hr, protime (PT), 14.4 seconds, prothrombin time (PTT), 28 seconds, and international normalized ratio (INR) of 1.1. Serum antinuclear antibodies (ANA), antineutrophil cytoplasmic antibodies (ANCA), and antiglomerular basement membrane antibodies (GBM) were all negative. Blood and sputum cultures remained negative, and the echocardiogram was normal. Chest X-ray (CXR) showed diffuse hazy opacities and right upper lobe partial atelectasis. Computed tomography (CT) scan of his chest demonstrated bilateral diffuse alveolar ground-glass opacities, which were greater at the lung apices with peripheral sparing and pulmonary consolidation in the right upper lobe posterior segment (Figures 1 and 2). Based on these imaging findings, the differential diagnosis included pulmonary edema and DAH.

Additional history revealed that the patient had been diagnosed with seizure disorder two years prior and was on levetiracetam 1000 mg twice daily. He had a similar episode of hemoptysis (5–10 mL) followed by a seizure that required hospitalization five months ago. Workup at that time was significant for CT chest also showing diffuse alveolar ground-glass opacities, a bronchoscopy revealing frank blood in airways, and a bronchoalveolar lavage that was without hemosiderin-laden macrophages. Rheumatology workup was normal except a positive ANA titer of 1 : 160 and a low positive anticardiolipin IgM of 20 IgM phospholipid units (MPL). Given that he had no signs or symptoms of systemic autoimmune illness, this serologic profile was considered clinically insignificant and nonspecific, especially with normal C3 and C4 levels. Of note, ANA was normal on subsequent admission. A genetic seizure panel was sent during the second admission, which returned positive for a pathogenic variant c.111111C > T (p.Gln371*∗*) in the SCP2 gene. His history was significant for mild intermittent asthma and seasonal allergies. Family history was notable for seizure disorder in both mother and brother.

**Figure 3:**
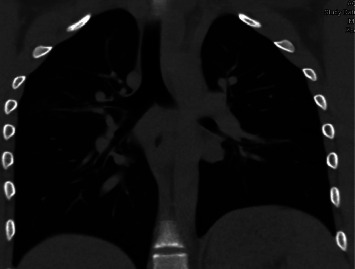
Post-extubation coronal view of CT chest scan showing the resolution of ground-glass opacities following diuretic therapy.

**Figure 4:**
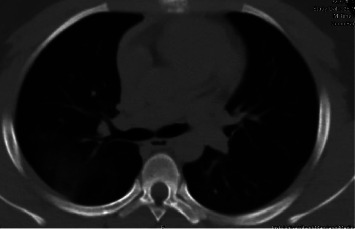
Axial view of CT chest scan showing a significant resolution of bilateral ground-glass opacities after treatment with diuretics.

With this additional history, a working diagnosis of recurrent seizure-induced hemoptysis was pursued. Given the concern of associated alveolar and interstitial edema, a trial of two doses of intravenous furosemide 40 mg, given eight hours apart, was initiated. The patient then showed considerable symptomatic improvement. Repeating CT chest post-extubation demonstrated near complete resolution of ground-glass opacities (Figures 3 and 4). Lung biopsy was deferred in view of symptomatic and imaging improvement, and he was discharged on day 5. Lung function testing and electroencephalography (EEG) post-discharge were within normal limits. He continues to remain symptom-free six months post-discharge.

## 3. Discussion

In the adult literature, a recent case series describes the presentation of post-ictal pulmonary hemorrhage in patients ranging 18–41 years of age [2]. Such patients have been treated with antiepileptics or steroids in previous scenarios [8]. Other case reports have used Alpha-1 adrenergic antagonists; however, the safety of those treatments has not been shown in pediatric literature [9, 10]. It has also been observed that pulmonary findings have regressed with supportive care [3], but in this case, we highlight the advantage of using diuretics. Whether or not diuretics help hasten recovery of a pediatric patient with this diagnosis remains a topic for further research.

During both hospitalizations, there was a negative infectious workup, normal echocardiogram, no history or signs of obvious trauma, and no visible foreign body on imaging or bronchoscopy (first episode). Vasculitis workup was nondiagnostic and there were no other systemic signs or symptoms of an autoimmune disorder. With this negative workup each time, the common causes of pediatric hemoptysis had been deemed unlikely. Imaging findings during both hospitalizations showed diffuse bilateral alveolar opacities. When assessed in the setting of unique hemoptysis recurrence in the setting of seizures only, the negative workup collectively pointed our team toward neurogenic or negative pressure pulmonary edema with consequent DAH as a diagnosis of exclusion. Moreover, while certain anticonvulsants like valproic acid have been reported to cause hemoptysis, our patient had been prescribed levetiracetam, making drug-induced DAH unlikely in this case. No other medical interventions outside of supportive care and seizure control were made during the admission, speaking for the success of diuretic therapy in the quickly ameliorating symptoms of neurogenic pulmonary edema.

This case report reiterates the importance of a tailored approach to the management of hemoptysis in different clinical conditions. Here, diuretic therapy was utilized to alleviate seizure-induced hemoptysis. However, larger studies and trials are needed to establish a safe and effective management strategy for this rare clinical entity. This case also describes a rarely reported diagnosis in the pediatric population, that of seizures inducing neurogenic pulmonary edema and causing diffuse alveolar hemorrhages with consequent hemoptysis. We also highlight the novel finding of the association of the SCP2 gene with this clinical presentation. Though associated with autosomal recessive forms of leukoencephalopathy and dystonia, the role of the SCP2 gene and its mutations in possible cell death following accumulation of toxic lipid metabolites and its systemic sequelae in other tissues such as the lung have not been clearly defined. More case reports and studies are required to understand the full spectrum of this condition and document if it can be helpful in predicting similar associations in the future, and this report may be of help in delineating future phenotypes of this mutation. Lastly, this case describes a rare but potentially life-threatening consequence of inadequate seizure control in pediatric patients, while offering a widely accessible treatment modality for it that led to full symptom resolution rapidly.
